# Winter diet of bats in working forests of the southeastern U.S. Coastal Plain

**DOI:** 10.1038/s41598-024-63062-3

**Published:** 2024-06-04

**Authors:** Santiago Perea, Colton D. Meinecke, Angela L. Larsen-Gray, Daniel U. Greene, Caterina Villari, Kamal J. K. Gandhi, Steven B. Castleberry

**Affiliations:** 1 Warnell School of Forestry and Natural Resources, University of Georgia, Athens, GA USA; 2 National Council for Air and Stream Improvement, Inc., Blacksburg, VA USA; 3 Weyerhaeuser Company, Environmental Research South, Columbus, MS USA

**Keywords:** Ecology, Genetics, Zoology, Environmental sciences

## Abstract

Working forests comprise a large proportion of forested landscapes in the southeastern United States and are important to the conservation of bats, which rely on forests for roosting and foraging. While relationships between bat ecology and forest management are well studied during summer, winter bat ecology remains understudied. Hence, we aimed to identify the diet composition of overwintering bats, compare the composition of prey consumed by bat species, and determine the potential role of forest bats as pest controllers in working forest landscapes of the southeastern U.S. Coastal Plain. During January to March 2021–2022, we captured 264 bats of eight species. We used DNA metabarcoding to obtain diet composition from 126 individuals of seven bat species identifying 22 orders and 174 families of arthropod prey. Although Coleoptera, Diptera, and Lepidoptera were the most consumed orders, we found that bats had a generalist diet but with significant differences among some species. We also documented the consumption of multiple insect pests (e.g., *Rhyacionia frustrana)* and disease vectors (e.g., *Culex* spp). Our results provide important information regarding the winter diet of bats in the southeastern U.S. Coastal Plain and their potential role in controlling economically relevant pest species and disease vectors.

## Introduction

The study of trophic resources is a key aspect of foraging ecology, providing a basic understanding of the relationships between consumers, resources, and the environment^1,2^. Insectivorous bats are important top-down regulators of arthropod populations^3–5^. Many bat species are characterized by a wide range of dietary preferences and can adapt to various land cover types, which enables them to adjust to changes in food availability throughout the year^6,7^. As highly mobile generalist consumers, insectivorous bats contribute to stabilizing and connecting local food webs in their ecosystem^8,9^. Additionally, they provide important ecosystem services by suppressing agricultural pests^10–13^, forest pests^4,14^, and vectors of parasites of humans^15,16^ and livestock^17,18^. For example, based on DNA metabarcoding of guano collected from roosts, Maslo et al.^13^ found that bats consumed ≥ 160 known agricultural pest species or disease vectors. Dietary studies focused on significant food resources and the effects of species interactions and communities are key to informing wildlife management decisions regarding species trophic position and population regulation^19^.

In most temperate zones, bats migrate or remain in torpor during winter^20^. However, milder climatic conditions of southern temperate latitudes, such as the Coastal Plain of the southeastern United States (U.S.), allow bats to remain active year-round or migrate from northern latitudes seeking warmer winter temperatures^21,22^. This region is especially relevant because the ability of Coastal Plain populations to maintain higher activity throughout the winter could translate into lower mortality associated with white-nose syndrome (WNS), an epizootic, infectious fungal disease caused by *Pseudogymnoascus destructans* (Pd). WNS has become the most serious threat to North American cave-dwelling bats, affecting overwintering bats by disrupting their torpor cycles and leading to increased energy expenditure and mortality rates. The fact that these are potential areas for remnant populations of species impacted by WNS in northern regions, combined with anthropogenic factors, such as wind energy development^21,23^, underscores the importance of understanding the ecology of bats in the southeastern Coastal Plain. Forests account for an important component of the Coastal Plain landscape, with > 86% of forests being privately owned^24^. Managed or working forests refer to forests that are actively maintained to achieve specific goals, such as the production of timber products, provision of recreational activities, creation of wildlife habitat, and carbon sequestration and storage. These forests are supported by economic incentives for sustainable management, which reduces the likelihood of their conversion to urban or agricultural land uses^25^. Working forests provide resources for a variety of wildlife species, including foraging and roosting resources for bats^26^ and, in turn, bats provide essential ecosystem services to forests, such as phytophagous insect control^3,27,28^.

The diet of North American bat species has traditionally been identified by morphological methods which involve identifying remains of prey in fecal samples^29–32^. However, identification of remains is difficult and biased toward hard-bodied insects, such as Coleoptera, which persist through digestion less degraded^33^. In recent years, DNA metabarcoding has contributed greatly to our understanding of predator–prey relationships, including the diet of bats in forests and agricultural systems. Metabarcoding enables elucidation of diet through simultaneous sequencing of a single DNA region from multiple constituent species of a complex sample^34^. Such studies have revealed predation of important pests for multiple agricultural commodities in North America^12,35,36^. For example, Boyles et al.^37^ valued the ecosystem services that insectivorous bats provide at $22.9 billion per year on agroecosystems across the United States. In addition, these advances in molecular techniques documented the consumption of insect vectors of human diseases^16,38^, including multiple arthropod-borne viruses (arboviruses). Overall, molecular techniques provide much information on the prey consumption preferences of bats. However, much remains to be understood in terms of diet overlap, resource distribution, and differences in availability across seasons (e.g., summer vs. winter).

**Figure 1:**
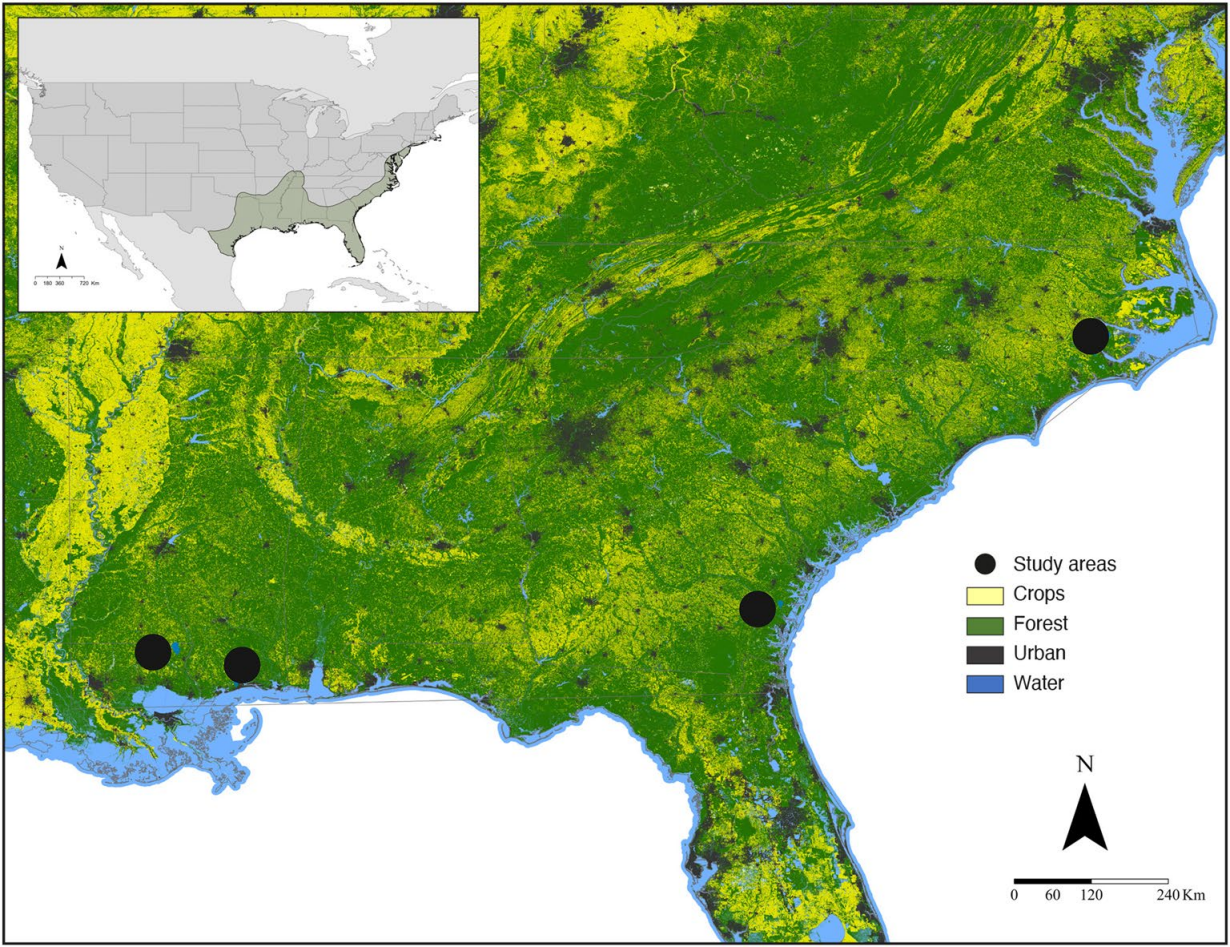
Location of study sites (circles) in the southeastern United States Coastal Plain where bat sampling was conducted from late-January to mid-March 2021–2022. Landscape cover types derived from a reclassification of The National Land Cover Database 2021.

To date, with the exception of Bernard et al.^39^, who evaluated the diet of cave-dwelling bat species captured outside caves during winter in Tennessee, United States, most molecular studies in North America focused on summer diet^12,33,38,40,41^. However, effective conservation decisions require a thorough understanding and assessment of trophic interactions among multiple species over time. Hence, it is imperative to understand the diet of bat communities throughout the year to obtain better estimates of ecological services^37,42^. Given the important representation of bats in forest vertebrate diversity, limited knowledge about dietary preferences during winter, and their roles as arthropod controllers (including pests of economic and health concern), we assessed the winter diet composition of bat communities on private, working forests of the southeastern U.S. Coastal Plain using DNA metabarcoding (Fig. 1). To better understand complex diet dynamics, our objectives were to (1) identify the diet composition of overwintering bats, (2) compare the composition of prey consumed by bat species, and (3) determine the potential role of forest bats as pest controllers in winter.

## Results

**Figure 2:**
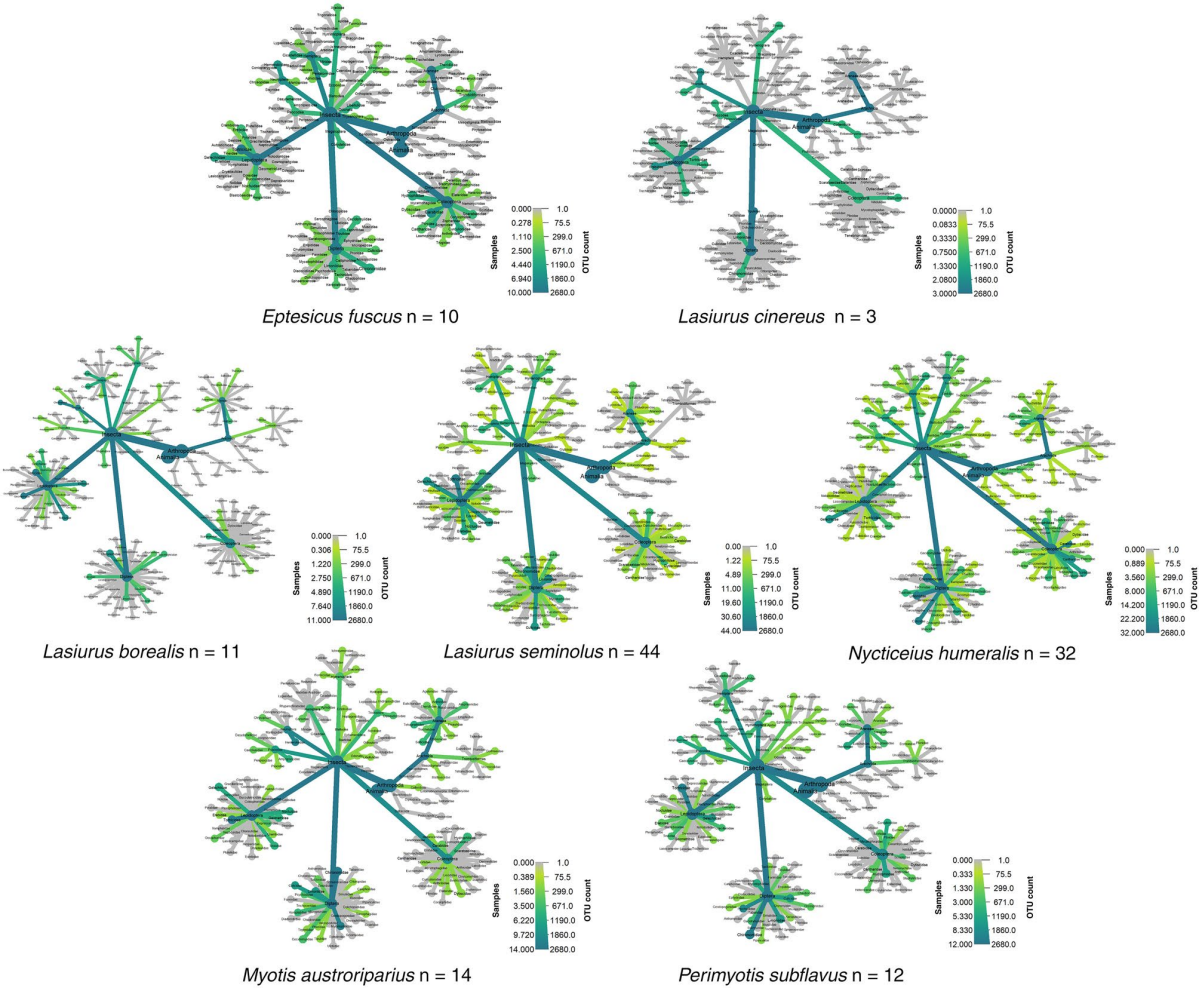
Winter diet including order, family, and genus of seven bat species in private, working forests of the southeastern U.S. Coastal Plain from late-January to mid-March 2021–2022. Colors represent number of samples and width of nodes represent number of Operational Taxonomic Unit (OTU) counts for each taxonomic level.

We captured 264 individuals of eight bat species from late-January to mid-March 2021–2022, collecting fecal samples from 209 individuals, from which we selected samples from 195 individuals. After bioinformatics processing and quality filtering, we obtained diet composition from 126 individuals of seven species (Table 1). None of the fecal samples from the two captured *Dasypterus intermedius* passed quality control. We obtained 2703 unique Operational Taxonomic Units (OTUs), 2127 (78.69%) of which were matched to sequences in the Barcode of Life Database v3 (BOLD) reference collection after pruning. These matches belonged to 22 orders, 174 families, and 422 genera or species (Supplementary Material 1). Within analyzed fecal samples, Coleoptera (n = 610 OTUs), Diptera (n = 684 OTUs), and Lepidoptera (n = 551 OTUs) were the most consumed orders (Supplementary Material 2). These three orders were the most consumed orders by all bat species except *Lasiurus cinereus* (Fig. 2; Table 1), which had a scarce representation of Coleoptera, although with a sample size of only three individual bats. For the remaining bat species, percentages varied among species such as *L. borealis* where 41.46% was based on Lepidoptera, to species such as *Eptesicus fuscus,* where 45.25% corresponded to Coleoptera, or *Perimyotis subflavus* with a preference for dipterans (49.21%) (Fig. 2, Table 1).

**Table 1:** Bats captured, number of fecal samples collected, number of samples analyzed, number of Operational Taxonomic Units (OTUs) for each bat species within insect orders, and bat species foraging strategies in private, working forest landscapes across four states (Georgia, Louisiana, Mississippi, and North Carolina) of the southeastern U.S. Coastal Plain from late-January to mid-March 2021–2022.

Species	Bats captured	Fecal samples	Samples analyzed	Coleoptera	Diptera	Lepidoptera	Other	Orders	Foraging strategy
*Lasiurus seminolus*	79	60	44	158	314	383	155	19	Edge-space aerial foragers
*Nycticeius humeralis*	75	54	32	281	371	132	216	22	Edge-space aerial foragers
*Myotis austroriparius*	41	37	14	202	186	132	68	17	Narrow-space, aerial-gleaning forager
*Perimyotis subflavus*	25	20	12	61	218	75	89	13	Edge-space aerial foragers
*Lasiurus borealis*	25	21	11	65	72	153	79	16	Edge-space aerial foragers
*Eptesicus fuscus*	14	12	10	200	77	92	73	16	Open and edge-space aerial foragers
*Lasiurus cinereus*	3	3	3	3	15	13	13	10	Open-space aerial foragers
*Dasypterus intermedius*	2	2	0	-	-	-	-	-	Open-space aerial foragers
Total	264	209	126	610	684	551	282	22	

Foraging strategies follow Norberg and Rayner^43^ and Denzinger and Schnitzler^44^.

Diet composition was dissimilar among species (Bray–Curtis: R = 0.27, *P* < 0.001). Additionally, we detected significant differences both in the dispersion of diet composition among species (Bray–Curtis: F_6,125_ = 2.01, r^2^ = 0.09, *p* = 0.001), and when performing the permutational dispersion test (F_6,125_ = 4.04, Nperm = 999, *p* = 0.002). Lastly, post-hoc pairwise multilevel comparisons revealed significant differences (*p* adj. < 0.05) in diet composition among *E. fuscus*/*Nycticeius humeralis* (F = 2.60, *p* adj. = 0.02), *L. borealis*/*N. humeralis* (F = 2.61, *p* adj. = 0.02), *L. cinereus*/*L. seminolus* (F = 1.65, *p* adj. = 0.04), *L. cinereus*/*N. humeralis* (F = 2.21, *p* adj. = 0.02), *L. seminolus*/*Myotis austroriparius* (F = 1.80, *p* adj. = 0.04), *L. seminolus*/*N. humeralis (*F = 4.32, *p* adj. = 0.02), and *M. austroriparius*/*N. humeralis* (F = 3.14, *p* adj. = 0.02). All other post-hoc pairwise multilevel comparisons did not show significant differences (*p* adj. > 0.05).

Bats consumed agricultural and forest pest species in five orders (Coleoptera [n = 12], Diptera [n = 2], Hemiptera [n = 5], Lepidoptera [n = 27], and Trombidiformes [n = 1]). Forest pests, including *Argyrotaenia pinatubana*, *Clepsis peritana*, *Hylobius pales*, and *Rhyacionia frustrana* were consumed by multiple bat species (Table 2). As for dipteran parasite vectors, we documented five genera of mosquitoes (Culicidae), highlighting mosquitoes of the genus *Culex*, including *C. nigripalpus*, *C. salinarius*, and *C. territans*, widely present in the diet of all bat species except for *L. cinereus* (Fig. 2, Table 2). Other known parasite vectors included two genera of black flies (Simuliidae), three genera of sandflies (Ceratopogonidae), and one genus of drain or sewer fly (Psychodidae) (Supplementary Material 3).

**Table 2:** Prey items Operational Taxonomic Units (OTUs) consumed by seven bat species captured in private, working forest landscapes across four states (Georgia, Louisiana, Mississippi, and North Carolina) of the southeastern U.S. Coastal Plain from late-January to mid-March 2021–2022.

Prey OTUs			Bat species						
Order	Family	Species	EPFU	LABO	LACI	LASE	MYAU	NYHU	PESU
Araneae	Salticidae	*Pelegrina montana*	2			5	4	7	1
	Theridiidae	*Robertus crosbyi*	2	1		5		**12**	
Coleoptera	Cantharidae	*Podabrus nothoides*	3	2		8	2	**22**	**4**
	Carabidae	*Oodes amaroides*	**4**			2	1	1	
	Carabidae	*Platynus cincticollis*	**5**			1	1	4	
	Carabidae	*Stenolophus ochropezus*	1					**10**	
	Curculionidae	Hylobius pales*	**5**		**2**	3	1	1	
	Curculionidae	Xylosandrus crassiusculus*		2		4	1	5	**3**
	Hydrophilidae	*Helocombus bifidus*	**5**	1		**19**	3	10	**5**
	Scarabaeidae	*Dyscinetus morator*		3	**1**	4		2	
	Scirtidae	*Contacyphon ochreatus*				6		**10**	2
Diptera	Chironomidae	*Chironomus df decorus*	**4**	1	1	8	**9**	**16**	2
	Chironomidae	*Chironomus harpi*	2	2	**3**	**16**	4	**18**	2
	Chironomidae	*Orthocladius oliveri*			1	1	**7**		
	Culicidae	*Culex nigripalpus*		1		14	**4**	**15**	2
	Culicidae	*Culex territans*	1	1		13	3	10	**4**
	Culicidae	*Culex salinarius*		1		**16**	2	**11**	**3**
	Limoniidae	*Erioptera caliptera*	4	1		6	1	9	**5**
	Psychodidae	*Psychoda alternata*	1			6		9	**5**
	Tipulidae	*Nephrotoma ferruginea*	3	1	**3**	5	1	4	
Hemiptera	Aphididae	*Eulachnus rileyi*	2	3		1		1	
Hymenoptera	Apidae	*Nomada subrutila*	3	1		11	2	8	3
	Xyelidae	*Xyela spp.*	3	**3**	1	**16**		**11**	3
Lepidoptera	Erebidae	Hypena scabra*	2	**3**	**2**	2		1	
	Erebidae	*Schrankia macula*		**3**	1	2			
	Geometridae	Eupithecia miserulata*		**4**		4			
	Geometridae	Orthonama obstipata*			1	1			1
	Geometridae	*Thysanopyga intractata*		**4**		**15**			
	Noctuidae	*Eupsilia vinulenta*	4	2		2	1	1	1
	Noctuidae	*Orthosia hibisci*		3	**2**	4			
	Noctuidae	*Sericaglaea signata*	2	**4**		5	2	2	2
	Plutellidae	Plutella xylostella*			**1**				
	Tineidae	*Nemapogon interstitiella*	1			1	**5**	1	2
	Tortricidae	Argyrotaenia pinatubana*	**7**	**7**	1	**15**	**9**	8	2
	Tortricidae	*Chimoptesis gerulae*	**5**	**6**		7	3	2	**3**
	Tortricidae	Clepsis peritana*	3	2		**24**	1	9	3
	Tortricidae	Rhyacionia frustrana*	**4**	**6**		**21**	**4**	**10**	**5**
Neuroptera	Chrysopidae	*Chrysoperla rufilabris*	2	**9**	**2**	**18**	3	1	1
	Hemerobiidae	*Hemerobius stigma*	4	2		2	**5**	1	
	Hemerobiidae	*Micromus posticus*	**4**	1	**2**	**20**	**9**	8	3
	Hemerobiidae	*Micromus subanticus*	2		**2**		1	1	1
Psocodea	Amphipsocidae	*Polypsocus corruptus*		1	1	6	**5**	4	**5**
Odonata	Libellulidae	*Trithemis dubia*	**8**						

Top ten items consumed by each bat species are highlighted in bold. Bat species codes: *Eptesicus fuscus* (EPFU), *Lasiurus borealis* (LABO), *Lasiurus cinereus* (LACI), *Lasiurus seminolus* (LASE), *Myotis austroriparius* (MYAU*), Nycticeius humeralis* (NYHU), and *Perimyotis subflavus* (PESU). The asterisk (*) denotes pest species.

## Discussion

Our results show a great variability (22 arthropod orders) in diet across bat species, highlighting the consumption mainly of Coleoptera, Diptera, and Lepidoptera. As expected, diet composition differed among bat species with different foraging strategies, but surprisingly also among species in similar foraging guilds. Our findings complement previous work conducted during summer indicating that bat diets vary seasonally^33,41,45^, which may depend on insect phenologies and weather conditions. Specifically in winter, seasonal prey limitations may lead to shifts towards more generalist behavior in several bat species, with changes in dietary composition and diversity compared to other times of the year and life stages of bats. Further, our results confirm the role of overwintering bat communities as consumers of agricultural and forest pests and potential arthropod vectors of human and animal diseases.

Traditionally, dietary preferences of insectivorous bats have been explained based on differences in their ecomorphologies and morphometric characteristics, with larger species feeding on larger insects or insects with more resistant exoskeletons^43,46^. The energetic cost–benefit of feeding on smaller insects compared to larger insects or insects with more resistant exoskeletons would lead to dietary selection based on the morphological characteristics of each bat species^47^. For example, it is often questioned whether species, especially large-body bats, can meet energy demands consuming small soft-body insects such as flies and mosquitoes^48^. However, availability and temporal variation of prey may lead to shifts in preferences towards more generalist diets. *Eptesicus fuscus*, the second largest of the seven species captured, is considered a coleopteran specialist e.g.,^30,49,50^. Recently, this assumption has been questioned, placing *E. fuscus* instead as generalist consumers in summer with preferences for Coleoptera when available^33,36,38^. In our study, a large portion of their diet was Coleoptera, but we found high dietary diversity, including many dipterans, possibly attributed to more dipterans in winter relative to other insect orders^39^. Flexible hunting strategies may allow bat species to adapt to different food availabilities by consuming prey that is abundant at the time, although of non-optimal sizes or other characteristics^51^. In contrast, the diet of *L. cinereus*, the largest species in our study and one of the largest species in North America, was comprised primarily of Diptera and Lepidoptera. Although our results should be interpreted with caution because of the small sample size (n = 3 individual bats), previous studies suggest that *L. cinereus* select large, soft-bodied insects (e.g., Lepidoptera and Neuroptera) and avoid small or hard-bodied insects (e.g., Coleoptera, Diptera, and Hemiptera)^52–54^. Most of the dipterans we documented in the diet were large crane fly species such as *Nephrotoma ferruginea* (Table 2), which supports a preference for large, soft-bodied prey.

The remaining bat species in our study are smaller and adapted to foraging along forest edges or within forests e.g.,^22,55,56^. *Lasiurus borealis* and *L. seminolus* share similar ecomorphologies, to the point that it is difficult to separate them by the characteristics of their echolocation calls or external morphology^57,58^. Both species have robust dentition like other Coleoptera specialists^47^. However, both ours and previous dietary analyses indicate that they consume a wide range of soft-bodied prey such as Diptera, Lepidoptera, and Neuroptera e.g.,^12,40,59^. The dietary differences identified between *N. humeralis* with *L*. *borealis* and *L. seminolus* could be due to the partitioning of selected prey within the same spaces and slight differences in ecomorphology and general external morphologies. The morphometrics and dentition of *N. humeralis* together with previous summer dietary analyses show flexibility in its diet, which allows it to eat a wide range of arthropods, from coleopterans to soft-bodied prey^30,47,60^. Our results confirm similar preferences in the diet during winter, where we observed high dietary diversity, distinguishing *N. humeralis* from other species. These findings are supported by the presence of OTUs from all 22 identified orders.

Previous works indicate that *M. austroriparius* and *P. subflavus* consume primarily soft body prey^30,59^. Using morphological dietary analyses, Feldhamer et al.^30^ found that both species consumed mainly trichopterans, suggesting a diet of soft-bodied species found predominantly above water. However, we observed numerous Coleoptera OTUs present in the diet of *M. austroriparius,* which highlights its dietary plasticity, consuming hard-bodied insects in winter. Differences between *M. austroriparius* with diets of *L. seminolus* and *N. humeralis* suggest a tendency towards a more specialized diet likely influenced by its forest-interior foraging strategies^22^. *Perimyotis subflavus* is among the smallest bats in North America^61^. Previous studies have noted that *P. subflavus* shows an opportunistic approach when foraging, exhibiting one of the most diverse diets in eastern North American bat species^59,62^. However, we found that *P. subflavus* consumed the second lowest number of orders, but a large proportion of dipterans, which concurs with previous research that documented frequent consumption of dipterans by *P. subflavus* in winter^39^. Disproportionate consumption of dipterans in winter compared to other seasons could be a consequence of a selection for small soft-bodied prey and a higher abundance of Diptera relative to other orders.

To our knowledge, our study is the first to document the consumption of agricultural and forest pests by winter bat communities in the southeastern U.S. Coastal Plain where intensive pine management and agriculture dominate the landscape. Among the most common forest pest species we documented in bat diets, *R. frustrana*, is an economically important pest of young pines, especially for loblolly pine (*Pinus taeda*), the preferred host species^63^. Our study coincided with the time period when *R. frustrana* typically emerges^64^, highlighting the importance of this moth to most bat species when availability is high. Additionally, *H. pales* was also widely consumed by most bat species in our study. *Hylobius pales* causes damage to young pine seedlings and is a vector of commercially damaging Ophiostomatalean “blue-stain” fungi such as *Leptographium* spp., which discolor and degrade the value of colonized wood^65,66^. Our research also reveals the consumption of various agricultural pests by bats, such as the moths *C. peritana* and *H. scabra*, which likely inhabit agricultural areas embedded within the working forest landscapes. While *H. scabra* was not the most frequently consumed pest nor found in large numbers, it was present in the winter diet of five bat species, including migratory species like *L. borealis* and *L. cinereus*^20^. *Hypena scabra* is a migratory moth, with most populations overwintering south of the midwestern U.S. Corn Belt^36,67^. Although it is generally of minor economic importance, this moth is one of the most common defoliating insects in alfalfa and soybean fields^68^. Consumption of overwintering populations of *H. scabra* in this ecoregion may provide a yet undocumented ecosystem service in controlling populations outside of the growing season and outside the major crop-producing areas of the Corn Belt. Overall, our findings suggest that consumption of agricultural and forest pests by bats in late winter and early spring could play a crucial role in minimizing damage during the subsequent growing season, highlighting the potential significance of bats as natural pest controllers in agricultural and forested landscapes.

Finally, we identified several species of flies and mosquitoes (Diptera) in winter diets that are recognized as threats to human health. Global concern about mosquitoes (Family Culicidae) stems from their significant impact on public health, attributed to their role as disease vectors. This impact extends to the transmission of multiple diseases (e.g., West Nile virus^69^, malaria^70^, dengue^71^, dog (*Canis lupus familiaris*) heartworm^72^, myxomatosis^73^, or avian malaria^74^) with far-reaching consequences for human societies, wildlife, and ecosystems. Our results reveal a diverse array of mosquito vectors, including species of the genera *Aedes* and *Culex*, common vectors of diseases such as West Nile virus. In addition, we identified malaria vectors, such as *Anopheles* mosquitoes, and specific cases of non-native mosquitoes, such as *Aedes japonicus*, implicated in the transmission and/or maintenance of arboviruses, both endemic to the region (e.g., West Nile virus) and exotic (e.g., Zika, dengue, and chikungunya)^75^. Although little known to date^42^, our results also demonstrated consumption of other dipterans that may pose a threat to wildlife, livestock, and poultry. For example, we confirmed consumption of Diptera such as black flies (Family Simuliidae), which are capable of transmitting pathogens, including protozoa and nematode worms to vertebrates, and are thus a veterinary concern, even if none of them cause disease in humans in North America^76^.

Identifying diet composition in overwintering bats and recognizing differences in prey consumption among species contribute valuable insights into the ecological role of bats in working forest landscapes. As these forests are crucial for remnant populations affected by WNS and migratory species affected by wind energy development, understanding winter bat foraging ecology becomes paramount. The potential role of forest bats as pest controllers during winter underscores the importance of managing working forests in ways that support the diverse dietary needs of the bat community. Our findings have a direct connection to economics and timber quality; for example, *R. frustrana* is known to have a drastic impact on pine growth, both in tree height and diameter^63^. Hence, proactive forest management practices that improve bat habitat conditions^77^, such as retention of hardwoods, trees with exfoliating bark, and cavity trees (live and dead) also increase their economic benefits. Additionally, our results show the role of bat communities outside forest boundaries consuming agricultural pests and other potential arthropod vectors of disease. Conservation efforts thus may consider ecological services provided by bats, including their ability to contribute to control of agricultural and forest pests and potentially limit the spread of disease vectors. We also emphasize the role of private lands conservation in promoting bat habitat and their consequent ecosystem services.

## Material and methods

### Study area

We conducted our study on private, working forest landscapes in late-January through mid-March, 2021–2022 in four states (Georgia, Louisiana, Mississippi, and North Carolina) (Fig. 1). Our study areas were characterized by a mosaic of forested landscapes with crop fields and areas with varying degrees of development^78^. We selected study areas > 3000 ha that consisted primarily of planted loblolly pine stands interspersed with riparian management areas (predominantly mature hardwood stands), roads, and wildlife openings. Management activities were typical of commercial forestry operations in the region, including clear-cutting at 20–35 years, mechanical and/or chemical site preparation, and planting 182–283 pine trees ha − 1^79^. Competing vegetation was temporarily suppressed through herbicide applications, prescribed fire, or mechanically, with most stands being thinned at least once. We defined January–March as the winter sampling season, as mean nighttime temperatures are lowest (typically < 10 °C) during this time in most of the Coastal Plain region^22^.

### Sample collection

We captured bats using a combination of single, double, and triple high net sets (Avinet Inc., Dryden, New York, U.S.; mesh diameter: 75/2, 2.6 m high, 4-shelves, 6–12 m wide) located along forest corridors, streams, under bridges, road ruts, and small ponds. We opened mist nets 30 min before sunset and left them open for 4–5 h, checking them every 10–15 min. We placed captured bats in individual clean paper bags and held them for 25–30 min to provide time for defecation^28^. After holding, we identified individuals to species, recorded sex, reproductive condition, forearm length (mm), and weight (g), and released them at the capture site. We collected 3–4 fecal samples from paper bags using sterile forceps, considering fecal samples from each individual bag as a single sample. We placed them into sterile 0.5 ml Eppendorf tubes (Eppendorf Inc., Enfield, Connecticut, U.S.) with 70% ethanol and stored them in coolers in the field and during transport to the laboratory. We stored samples at − 80 °C prior to DNA extraction.

### Ethics statement

Field research followed U.S. Fish and Wildlife Service’s (USFWS) WNS Decontamination Guidelines^80^ and recommended strategies to reduce risk of transmission of SARS-CoV-2 from humans to bats^81^. All capture and handling techniques were approved by the University of Georgia Animal Care and Use Committee #A2019 11-017-Y3- 168 A0), in compliance with the ARRIVE guidelines, and were consistent with guidelines published by the American Society of Mammalogists^82^. We obtained federal (#ES60238B) and state permit collections (Georgia Scientific Collection Permit #1000598963, Mississippi Scientific Collection Permit #0210211, Louisiana Scientific Collection Permit #WDP-22-002, and North Carolina Scientific Collection Permit numbers: Endangered Species Permit #21-ES00643 and NC Wildlife Collection License #22-SC01323).

### DNA extraction, library preparation, and sequencing

We ground each fecal sample in a 1.5 mL microcentrifuge tube using a micropestle to homogenize feces and increase surface area, then centrifuged. We then aspirated and discarded the ethanol, and samples were allowed to dry briefly under sterile conditions. We extracted DNA from up to 250 mg of each sample using a Qiagen QIAmp DNA Stool Mini Kit following the manufacturer's protocol (Qiagen, Germantown, Maryland, U.S.) with minor modifications. We prepared at least one blank extraction from each extraction kit and used it as a negative control in downstream analyses. We assessed quality and concentration of DNA extracts using a NanoDrop™ One microvolume UV–Vis spectrophotomter (Thermo Fisher Scientific, Waltham, Massachusetts, U.S.) prior to library preparation. We stored all DNA extracts at − 20 °C until amplification. We selected the number of samples to be sequenced based on the quality and concentration of DNA and abundance of samples for each species, avoiding selecting multiple samples of a species from the same site and night when possible.

A segment of the cytochrome c oxidase subunit I (COI) was amplified using the ANML primer pair, LCO1490 and CO1-CFMRa^83,84^. The ANML primers demonstrate preferential binding to arthropod COI and enhance the representation of arthropod taxa relative to mammalian and avian predator DNA^84^. We modified primers to contain 5′ overhang sequences required for Illumina library preparation and were synthesized by Integrated DNA Technologies (Coralville, Iowa, U.S.). The PCR reaction mixture consisted of 12.5 uL KAPA HiFi HotStart ReadyMix (Kapa Biosystems, Cape Town, South Africa), 2.5 uL of each primer (2.0 uM), 5 uL genomic DNA, and 2.5 uL molecular-grade water, for a final volume of 25 uL. Amplification reactions began with an initial denaturation of 95 °C for 3 min, 25 cycles at 95 °C for 30 s, 55 °C for 30 s, and 72 °C for 30 s, followed by a final extension at 72 °C for 5 min^84^. We checked all PCR products for successful amplification using gel electrophoresis, and we retained any samples that displayed at least a faint band at approximately 180 bp for further library preparation. We submitted sample amplicons to the Georgia Genomics and Bioinformatics Core (GGBC) for the remaining library preparation steps and sequencing on the Illumina NextSeq 2000 (Illumina, San Diego, California, U.S.). We generated paired-end reads (i.e., each amplicon was sequenced twice, once in each direction) at a length of 301 bp using the NextSeq 2000 P3 reagent kit (300 cycles, Illumina).

### Bioinformatic analyses

We demultiplexed reads by GGBC and received in FASTQ format. We performed all DNA sequence processing using the AMPtk pipeline^85^. We trimmed sequences to remove low-quality (< Q20) bases and primers and merged them. We then filtered reads for overall quality, dereplicated them to identify unique sequences, sorted each by abundance, and grouped each into OTUs at a 97% identity threshold using UPARSE^86,87^. We then applied the LULU algorithm to identify and correct errors^88^. Finally, we assigned taxonomic identities to OTUs using USEARCH^89^. We based taxonomic identities on the consensus agreement among three independent comparisons of sequences to the Barcode of Life Database v3 (BOLD) using global alignment, SINTAX, and UTAX algorithms^85^.

### Statistical analyses

We conducted all analyses and visualizations in R 4.1.1^90^. We analyzed diet composition by bat species and overlap among species using OTUs with assigned taxonomy. We first identified and filtered out rare taxa, defined as those with fewer than 10 reads across all samples^39^, and samples with fewer than 1,000 reads using the Phyloseq package (version 1.38.0^91^). We examined diet composition using heat trees constructed with the Metacoder package, which display taxa that were identified in samples and their lineage (version 0.3.6^92^). Specifically, we developed a single heat tree for each bat species, representing all insect families consumed by that species and individual heat trees for the three dominant insect orders consumed. Then, we used the VEGAN package (version 2.6.4^93^) to test for variations in prey composition among bat species by Analysis of Similarity (ANOSIM) and Permutational Multivariate Analysis of Variance (PERMANOVA) tests with 999 permutations^94^. Because PERMANOVA can sometimes be affected by non-homogeneity of dispersion for unbalanced sampling schemes, we also performed a permutational dispersion test^94^. Lastly, we performed post-hoc pairwise multilevel comparisons using the pairwise Adonis package with Bonferroni adjustment (version 0.4.1^95^) to determine differences among species.

### Supplementary Information

Supplementary Legends.

Supplementary Information 2.

Supplementary Figure 1.

Supplementary Information 4.

Supplementary Table 1.

## Data Availability

The dataset generated during and/or analyzed during the current study are available from the corresponding author on reasonable request.
