# Author Correction: Winter diet of bats in working forests of the southeastern U.S. Coastal Plain

**DOI:** 10.1038/s41598-024-65259-y

**Published:** 2024-06-21

**Authors:** Santiago Perea, Colton D. Meinecke, Angela L. Larsen‑Gray, Daniel U. Greene, Caterina Villari, Kamal J. K. Gandhi, Steven B. Castleberry

**Affiliations:** 1 Warnell School of Forestry and Natural Resources, University of Georgia, Athens, GA USA; 2 National Council for Air and Stream Improvement, Inc., Blacksburg, VA USA; 3 Weyerhaeuser Company, Environmental Research South, Columbus, MS USA

Correction to: *Scientific Reports* 10.1038/s41598-024-63062-3, published online 04 June 2024

The original version of this Article contained errors.

In the Introduction section, the fungal disease associated with the white-nose syndrome was misspelled.

“This region is especially relevant because the ability of Coastal Plain populations to maintain higher activity throughout the winter could translate into lower mortality associated with white-nose syndrome (WNS), an epizootic, infectious fungal disease caused by *Pseudogtmnoascus destructans* (Pd).”

now reads

“This region is especially relevant because the ability of Coastal Plain populations to maintain higher activity throughout the winter could translate into lower mortality associated with white-nose syndrome (WNS), an epizootic, infectious fungal disease caused *by Pseudogymnoascus destructans* (Pd).”

Furthermore, in the Results section

“We captured 264 individuals of eight bat species from late-January to mid-March 2021–2022, collecting fecal samples from 206 individuals, from which we selected samples from 195 individuals. After bioinformatics processing and quality filtering, we obtained diet composition from 125 individuals of seven species (Table 1).”

now reads,

“We captured 264 individuals of eight bat species from late-January to mid-March 2021–2022, collecting fecal samples from 209 individuals, from which we selected samples from 195 individuals. After bioinformatics processing and quality filtering, we obtained diet composition from 126 individuals of seven species (Table 1).”

The original Article has been corrected.

