# Supplementary material for: Winter diet of bats in working forests of the southeastern U.S. Coastal Plain
Source: Sci Rep. 2024 Jun 4;14:12778. doi: 10.1038/s41598-024-63062-3 (PMC11150266; doi:10.1038/s41598-024-63062-3)
Supplement: Supplementary file 1 — Supplementary Legends. [file 41598_2024_63062_MOESM1_ESM.docx]

**Supplementary material**

Supplementary Material 1. Arthropods consumed by winter bat communities, listed by order, in private, working forests of the southeastern U.S. from late-January to mid-March 2021-2022.

Supplementary Material 2. Figure 1. Family, genus, and species diversity for the three insect orders (Coleoptera, Diptera, and Lepidoptera) most abundant in the diet of wintering bat communities in private, working forests of the southeastern U.S. from late-January to mid-March 2021-22. Colors represent number of samples and width of the nodes represents the number of reads for each taxonomic level.

Supplementary Material 3. List of agricultural and forest pests and arthropod disease vectors consumed by overwintering bat communities on private, working forests in the southeastern U.S. from late-January to mid-March 2021-2022.
