# Supplementary figures and images for: Winter diet of bats in working forests of the southeastern U.S. Coastal Plain
Source: Sci Rep. 2024 Jun 4;14:12778. doi: 10.1038/s41598-024-63062-3 (PMC11150266; doi:10.1038/s41598-024-63062-3)

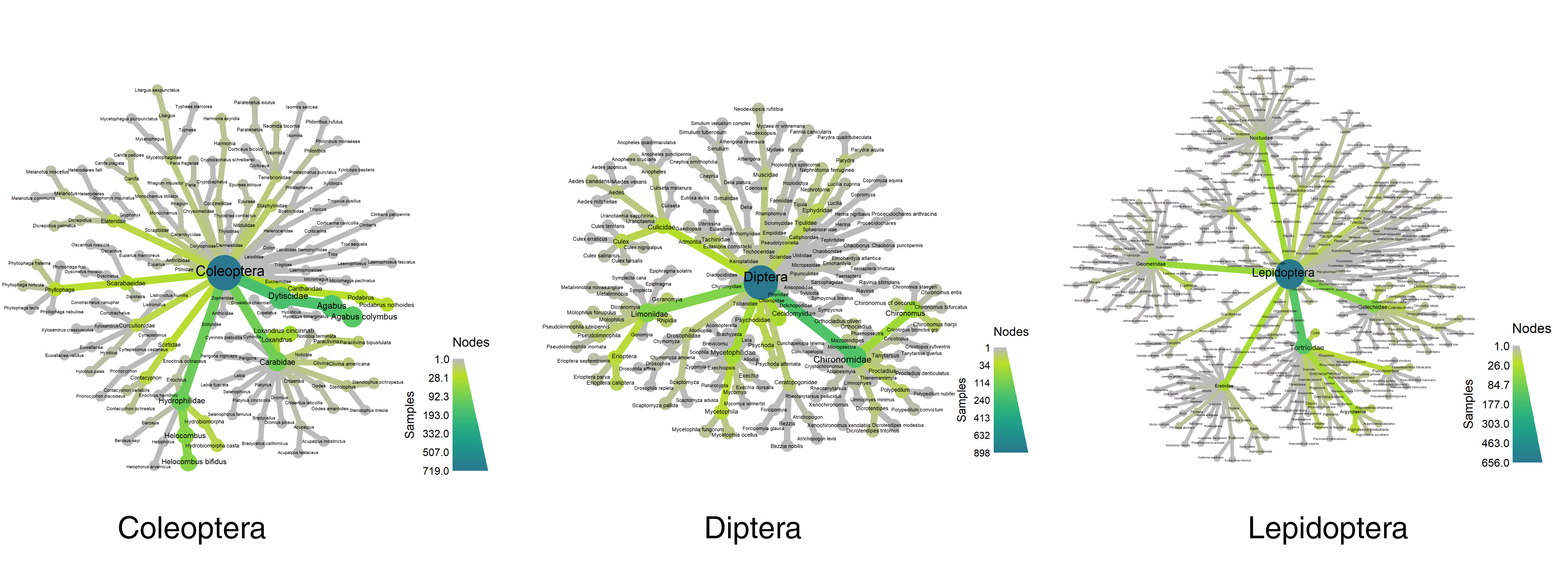

Supplement: Supplementary file 3 — Supplementary Figure 1. [file 41598_2024_63062_MOESM3_ESM.png]
