# Supplementary material for: Winter diet of bats in working forests of the southeastern U.S. Coastal Plain
Source: Sci Rep. 2024 Jun 4;14:12778. doi: 10.1038/s41598-024-63062-3 (PMC11150266; doi:10.1038/s41598-024-63062-3)
Supplement: Supplementary file 5 — Supplementary Table 1. [file 41598_2024_63062_MOESM5_ESM.docx]

Supplementary table 1. Total nights sampled, dates, and total number of bat species captured in private, working forest landscapes across four states (Georgia, Louisiana, Mississippi, and North Carolina) of the southeastern U.S. Coastal Plain from late-January to mid-March 2021-2022. Bat species codes: *Dasypterus intermedius* (DAIN), *Eptesicus fuscus* (EPFU), *Lasiurus borealis* (LABO), *Lasiurus cinereus* (LACI), *Lasiurus seminolus* (LASE), *Myotis austroriparius* (MYAU), *Nycticeius humeralis* (NYHU), and *Perimyotis subflavus* (PESU).

| **State** | **Nights** | **Dates** | **DAIN** | **EPFU** | **LABO** | **LACI** | **LASE** | **MYAU** | **NYHU** | **PESU** | **Total** |
| --- | --- | --- | --- | --- | --- | --- | --- | --- | --- | --- | --- |
| Georgia | 18 | 15 February 2024 / 07 March 2024 | 2 | 7 | 4 | 0 | 51 | 10 | 26 | 20 | **120** |
| Louisiana | 14 | 25 January 2024 / 11 Feb 2024 | 0 | 0 | 3 | 0 | 7 | 2 | 0 | 0 | **12** |
| Mississippi | 13 | 19 February 2023 / 13 March 2023 | 0 | 1 | 2 | 0 | 15 | 0 | 36 | 0 | **54** |
| North Carolina | 11 | 16 February 2024 / 07 March 2024 | 0 | 6 | 16 | 3 | 6 | 29 | 13 | 5 | **78** |
